# Subacute combined degeneration of the spinal cord masking motor neuron disease: a case report

**DOI:** 10.1186/s13256-019-2256-8

**Published:** 2019-11-18

**Authors:** Paula Loveland, Aaron Wong, Vinojini Vivekanandam, Wen Kwang Lim

**Affiliations:** 1 Department of Medicine and Aged Care, The Royal Melbourne Hospital, Parkville, VIC Australia; 2 Department of Aged Care, Austin Hospital, Heidelberg, VIC Australia; 3 Department of Neurology, The Royal Melbourne Hospital, Parkville, VIC Australia; 4 University of Melbourne, Parkville, VIC Australia

**Keywords:** Vitamin B 12, subacute combined degeneration, motor neuron disease, schizophrenia

## Abstract

**Background:**

Subacute combined degeneration of the spinal cord is a potentially reversible myelopathy typically associated with vitamin B12 deficiency. There is predominant involvement of spinal cord posterior and lateral tracts, and manifestations include peripheral paraesthesia, impaired proprioception, gait disturbance, neuropathy and cognitive changes. Motor neuron disease (MND) is an unremittingly progressive neurodegenerative disorder involving upper and lower motor neurons with an average prognosis of 2–3 years. The diagnosis is clinical and may be supported by electromyography. A subset of MND occurs concurrently with frontotemporal dementia (FTD-MND) and may be initially misdiagnosed as a primary psychotic disorder.

**Case presentation:**

We describe a 57-year-old Caucasian woman who presented with confusion and dysarthria. Low vitamin B12 levels and MRI findings led to an initial diagnosis of subacute combined degeneration of the spinal cord. Despite treatment, persistent dysarthria and presence of both upper and lower motor neuron signs on clinical examination prompted further assessment. Electromyography supported the diagnosis of MND. Comorbid chronic paranoid schizophrenia complicated the diagnostic process. We discuss overlapping features between B12 deficiency and MND as well as the neuropsychiatric overlap of B12 deficiency, FTD-MND and chronic schizophrenia.

**Conclusions:**

Firstly, variability in neurocognitive and imaging manifestations of B12 deficiency can limit delineation of other pathologies. Failure to improve following correction of nutritional deficiencies warrants further investigation for an alternate diagnosis. Secondly, re-evaluation of patients with comorbid mental health conditions is important in reaching timely and accurate diagnoses.

## Background

Subacute combined degeneration of the spinal cord is a potentially reversible myelopathy typically associated with vitamin B12 deficiency. There is predominant involvement of spinal cord posterior and lateral tracts, and manifestations include peripheral paraesthesia, impaired proprioception, gait disturbance, neuropathy and cognitive changes [1]. Motor neuron disease (MND), its commonest subtype being amyotrophic lateral sclerosis (ALS), is an unremittingly progressive neurodegenerative disorder involving upper and lower motor neurons with an average prognosis of two to three years [2]. The diagnosis is clinical and may be supported by electromyography (EMG). Neuroimaging findings in both conditions are insensitive, often unremarkable, or as rarely described they may be similar or even indistinguishable [3, 4]. We highlight the diagnostic challenges posed by coexistence of vitamin B12 deficiency and another neurological pathology, particularly MND, as there are overlapping clinical features common to both conditions.

The importance of a high index of suspicion in patients with cognitive and psychiatric conditions is also reinforced by our case, as such patients are vulnerable to missed or delayed diagnoses. ‘Diagnostic overshadowing’, or misattribution of disease manifestations to that of a known psychiatric condition, is commonly described in the literature, as are atypical disease presentations due to a variety of factors [5].

We also discuss our patient’s case in light of the subset of MND patients that manifest psychotic symptoms as part of the MND with frontotemporal dementia (FTD-MND) syndrome, of which the C9ORF72 mutation is the commonest abnormality in familial cases. Recent genetic studies also suggest a possible link between MND and chronic psychosis without dementia.

## Case presentation

A 57-year-old Caucasian woman with a background of treatment-resistant schizophrenia for over 30 years, stable on clozapine, was admitted after being found confused, dysarthric and dehydrated at home, having been uncontactable for several days. Her medical history included unprovoked pulmonary emboli on warfarin and emphysema, and her family history was significant for one brother with schizophrenia and one unaffected sister, but no known family members with MND or dementia-type diagnoses.

Confusion improved following hydration, but she was unable to recall recent events. Paranoia and distrust of staff were consistent with her chronic delusions and limited engagement with assessments. Neurological examination revealed tongue atrophy, increased upper and lower limb tone with hyperreflexia bilaterally, left upper limb and lower limb weakness and proprioception deficits without ataxia.

**Fig. 1:**
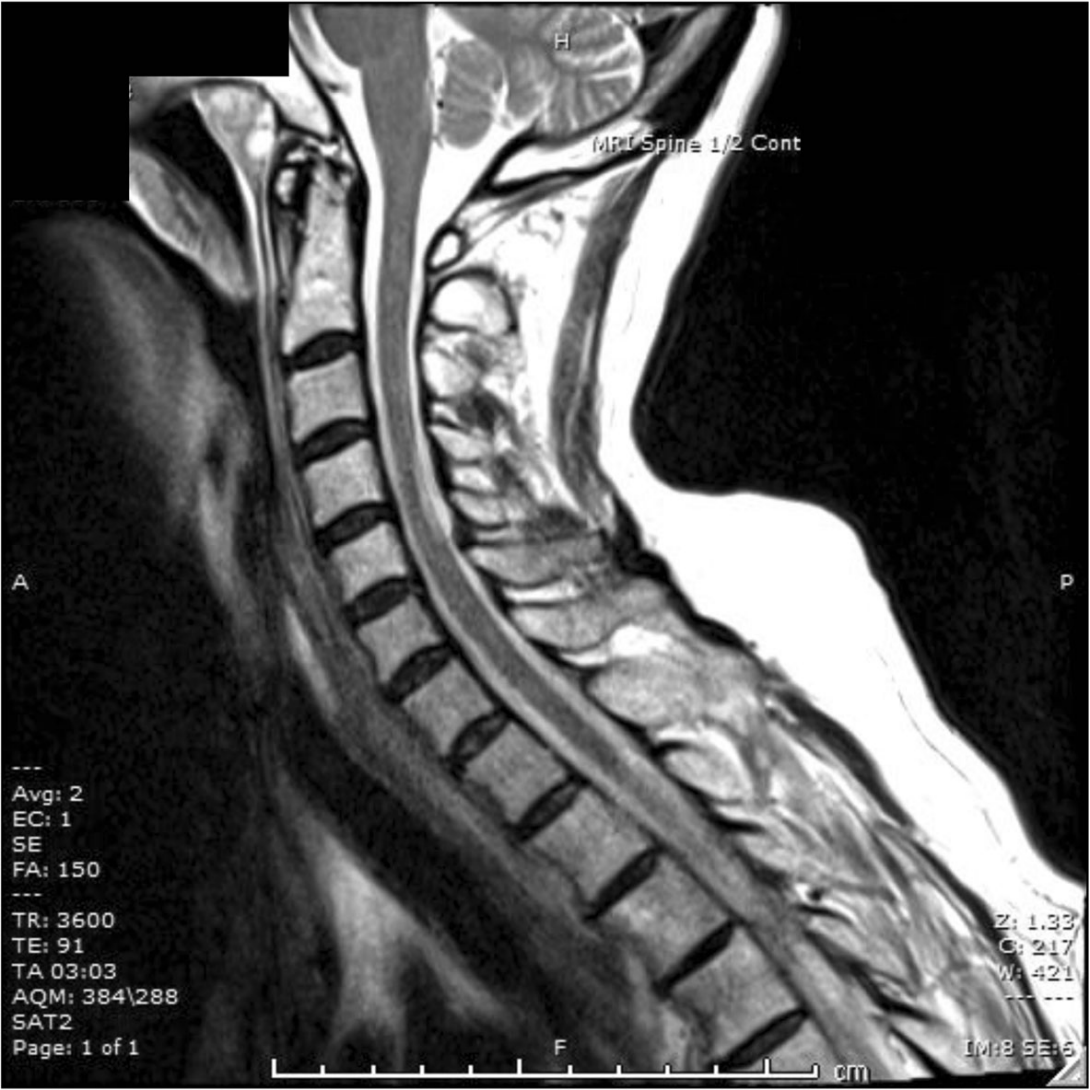
T2-weighted magnetic resonance imaging (MRI) of the spine demonstrating abnormal high T2 signal involving the dorsal column of the cervical cord

On admission, full blood examination, electrolytes and renal function were normal. Creatinine kinase was elevated at 882 IU/L (reference: 30–70 IU/L), vitamin B12 level was low at 69 pmol/L (140–650 pmol/L) and folate was normal at 31.5 nmol/L (> 7.0 nmol/L). Computed tomography (CT) scan of the head, MRI diffusion weighted imaging of the brain and electroencephalogram were unremarkable. Subsequent investigations detected other micronutrient deficiencies (iron, copper, zinc) but negative coeliac and pernicious anaemia screens, bland cerebrospinal fluid, and negative serology for relevant infections and anti-neuronal antibodies. Magnetic resonance imaging (MRI) showed abnormal high T2 signal involving the dorsal column of the cervical cord (Fig. 1), consistent with subacute combined degeneration of the spinal cord.

On day 10 she was transferred to our rehabilitation unit. Dysarthria necessitating written communication aids persisted, despite intramuscular B12 replacement. Examination further identified profound tongue wasting with intermittent fasciculations and brisk jaw-jerk reflex. Limb reflexes were brisk and power was decreased in the left upper limb only. She had no sensory or gait abnormalities. Chronic paranoid delusions continued but were non-distressing. Her mini-mental state examination scored 27/30 points, and Frontal Assessment Battery 13/18 points, before discharge. The combination of bulbar weakness, mixed upper and lower motor neuron signs and failure to improve despite B12 replacement prompted EMG. Widespread chronic denervation in upper and lower limbs and genioglossus alongside active denervation and fasciculation supported a diagnosis of MND. Interestingly, nerve conduction studies demonstrated no evidence of a sensorimotor neuropathy.

Our patient commenced treatment with riluzole and was referred to a multidisciplinary progressive neurological diseases service. Her disease progressed and she unfortunately died a number of months later.

## Discussion and conclusions

Our case demonstrates several important clinical lessons. Firstly, the diagnostic difficulties posed by coexistence of vitamin B12 deficiency and another neurological pathology, particularly motor neuron disease (MND). Variability in neurocognitive manifestations of B12 deficiency can limit delineation of other pathologies clinically. Clinicians should also be aware of non-specific investigation findings including the limitations of imaging findings in the setting of B12 deficiency and motor neuron disease (MND). Secondly, we present a reminder of the importance of careful examination and re-assessment where co-morbid schizophrenia or other mental illness may cloud the initial diagnosis. To our knowledge, this is the first report highlighting this combination of diagnostic challenges.

Neurological and cognitive effects of B12 deficiency, including subacute combined degeneration of the spinal cord, are highly variable [6]. Dysarthria is atypical, however, and its prominence in our patient eventually triggered investigation for an alternate diagnosis. Prognosis is excellent if B12 deficiency is corrected, although neurological deficits may persist. In contrast, MND is a progressive disorder involving upper and lower motor neurons, often with prominence of bulbar weakness. It manifests as weakness in the limbs, bulbar and respiratory muscles, usually with asymmetrical onset. The diagnosis is clinical and may be supported by EMG findings. Sensory function, ocular muscles, continence and cognition are typically spared [2].

Although our patient was diagnosed with MND, initial proprioceptive disturbance which resolved following B12 replacement may reflect concurrent subacute combined degeneration. This may have been sequelae of her paranoid schizophrenia limiting her food repertoire, compounded by bulbar dysfunction affecting nutritional intake. We noted that previous vitamin B 12 levels had been normal.

Neuroimaging findings in both B12 deficiency and MND are non-specific and may be absent or very similar. Both conditions have been associated with MRI hyperintensity on T2 weighted images of the brain and cervical cord, with the anatomical distinction of the dorsal columns and the pyramidal tracts being typically abnormal in B12 deficiency and MND respectively [1, 6]. However subacute combined degeneration of the spinal cord is documented to rarely result in demyelination of pyramidal and spinocerebellar tracts [7]. Another case report documented corticospinal tract hyperintensity on MRI scan of the brain of a B12-deficient patient whose neurocognitive symptoms improved with B12 replacement therapy, hence considered to be mimicking MND [4].

Clinically, vitamin B12 deficiency has been described to masquerade as antipsychotic-resistant psychosis [8]. Vitamin B12 supplementation with folate has been proposed as a method of improving negative symptoms in certain schizophrenic patient groups [9]. However a meta-analysis more recently reported that vitamin B12 levels in patients with schizophrenia were not lower, but rather higher, overall, than the control group [10]. The authors hypothesise that perhaps patients with chronic psychosis were receiving more frequent monitoring and correction of nutritional status.

A subset of MND occurs concurrently with frontotemporal dementia (FTD-MND) and may be initially misdiagnosed as a primary psychotic disorder. Its phenotype can include psychotic features, atypical MRI findings and, less commonly, primary progressive aphasia or parkinsonism [3]. The *C9ORF72* mutation with hexanucleotide expansion is the commonest known associated gene in familial cases of FTD-MND and can occur sporadically. While her first-degree relative with schizophrenia is proposed to be an important predictive factor, our patient’s history of over 30 years of antipsychotic-responsive symptoms preceding MND onset, however, is not in keeping with reported MND onset within 10 years of psychosis in FTD-MND [11]. Mutation testing for *C9ORF72* was considered given her comorbid psychiatric diagnosis, however our patient deteriorated before testing could be discussed.

More recently, a genetic link between schizophrenia and ALS has been suggested by studies that have found the *C9ORF72* expansion in first degree relatives with primary psychotic conditions without dementia and a significant genetic correlation between ALS and schizophrenia in a genome-wide study of over 100,000 people [12, 13]. This has increased interest in exploring overlapping management strategies for both conditions. The potential that neuroprotective effects of schizophrenia pharmacotherapy may protect against manifestations of ALS has also been hypothesised previously [14]. The relationship between neurodegenerative and neuropsychiatric conditions requires further delineation and characterisation.

Our case demonstrates the diagnostic challenges in the presence of concomitant and overlapping neurological conditions with co-existing psychiatric illness. The phenomenon of incorrectly attributing physical symptoms to a psychiatric condition is described in the literature as diagnostic overshadowing [5]. A number of case reports describe missed diagnoses due to comorbid psychosis [15, 16]. To our knowledge, we report the first case of ALS/MND and vitamin B12 deficiency causing subacute combined degeneration in the context of schizophrenia. Other important factors delaying clinical diagnoses in patients with psychiatric conditions include communication difficulties, symptoms being attributed to medication side effects and delay in seeking medical attention [17, 18]. Clinical vigilance and re-evaluation are key aspects in facilitating accurate and timely diagnosis in this vulnerable cohort of patients.

## Data Availability

All the data supporting our findings is contained within manuscript.

## Abbreviations

ALS: Amyotrophic lateral sclerosis
CT: Computed tomography
EMG: Electromyography
FTD: Frontotemporal dementia
MND: Motor neuron disease
MRI: Magnetic resonance imaging
